# Model‐Based Cost‐Effectiveness of Direct Restorations: Amalgam Dominates

**DOI:** 10.1111/cdoe.70050

**Published:** 2025-12-21

**Authors:** O. Bailey, S. J. Stone, G. Taylor, L. Ternent, C. R. Vernazza

**Affiliations:** ^1^ School of Dental Sciences Newcastle University Newcastle upon Tyne UK; ^2^ Population Health Sciences Institute Newcastle University Newcastle upon Tyne UK

**Keywords:** amalgam, caries, composite, decision analytic modelling, economics, health service

## Abstract

**Objectives:**

A phase‐down of dental amalgam use has been mandated and the feasibility of its phase‐out in England by 2030 is being explored. Amalgam use in English National Health Service (NHS) care still predominates for posterior restorations, though access to this care is increasingly limited. The objective of this study was to quantify the relative long‐term costs and consequences of amalgam versus composite direct posterior restorations in adult permanent teeth in the English NHS setting.

**Methods:**

A microsimulation model of restoration failure and reintervention was constructed and parameterised in TreeAge Pro, based on a review of the literature. It extrapolated costs and outcomes of directly restoring cariously‐cavitated lower premolar teeth in 10 000 18‐year‐old English NHS patients with amalgam and composite restorations over a lifetime‐horizon. Discounting of 3.5% was applied to costs and outcomes. An extended medical‐sector perspective with societal considerations was taken. Deterministic and probabilistic sensitivity analyses were performed.

**Results:**

Amalgam robustly dominated conventional and bulk‐fill composite restorations, being less costly over a lifetime from patient (£70) and funder perspectives (£34), with the restoration and tooth surviving significantly longer (4‐years; 12‐years non‐discounted). Amalgam also incurred reduced numbers of visits (1), treatment time (43‐min), and laboratory costs (£8) for fixed and removable prosthodontics over a lifetime. Time until a direct restoration was no longer possible was significantly higher for amalgam than composite (6‐years; 17‐years non‐discounted).

**Conclusions:**

The model showed good internal and external validity, accurately predicting tooth survival following restoration in relation to long‐term NHS claims data. Without considerable educational change to upskill clinicians and health service change, an amalgam phase‐out in England will likely have significant lifelong impacts on restoration and tooth survival and costs for all stakeholders, whilst reducing societal productivity and exacerbating already existing issues of limited access to care and socio‐economic inequalities.

## 1 Introduction

A phase‐down of dental amalgam use has been mandated on environmental grounds and the feasibility of its phase‐out in England by 2030 is being explored [1] following the Minamata Convention on Mercury [2]. A new EU‐specific regulation, to which the UK is not beholden, has mandated an amalgam phase‐out [3]. An estimated 1.1 billion direct dental restorations were placed globally in 2014 [4]. National Health Service (NHS) amalgam expenditure in 2015–2016 was crudely estimated at £200–300 million (K. Carr, personal communication, 2018).

Use of amalgam in many other European, primarily affluent, countries was very low prior to the EU phase‐out and the extra costs associated with composite were borne by the patients [5]. Amalgam use in posterior teeth under English NHS care still predominates [6], despite access becoming increasingly limited [7]. Composite resin (composite) has been described as the only reasonable alternative material in the required time frame [8, 9] though acknowledged as unable to replace amalgam in all situations [9]. There are a multitude of composite options with different characteristics which behave differently, for example paste and flowable formulations and conventional and bulk‐fill varieties [10, 11, 12]. Though glass ionomer cements and their derivatives have increasing levels of clinical data which show promise, they are still primarily short‐term, used for limited indications and are not commonly used in NHS primary care [6, 9].

Randomised controlled clinical trial data, though dated, heavily favour amalgam in terms of clinical effectiveness [13]. Composite can be very successful over the long‐term even in large cavities, but the data supporting this is retrospective and from expert operators commonly using optimal techniques (e.g., sectional matrices and multiple matrices per cavity) [14, 15, 16]. These techniques are rarely used in UK NHS primary care [6, 16].

Whilst there is a relatively large body of effectiveness data, economic data comparing amalgam and composite or assessing the effect of an amalgam phase‐down or ‐out are significantly more limited [16]. The World Health Organisation said that, ‘systematic studies on the economic and social costs and benefits of quality mercury‐free materials have not yet been published’ [17]. Economic evaluations can be based on cost and outcome data gathered from a clinical trial for the period of the trial alone, or can extrapolate findings over a lifetime using modelling techniques. Extrapolation attempts to quantify the differences in costs and outcomes between restorations over a lifetime, but carries more uncertainty [16].

When assessing the cost‐effectiveness of amalgam and amalgam alternatives, previous models have inevitably simplified the restorative cycle as the reintervention data is very limited [16, 18]. They have also used data sources to form the model and inform how restorations fail which are not relevant to the NHS primary care perspective. For example, a recent Canadian model assumes restorations are replaced by the same restoration each time they fail with the same survival [18]. Another German model assumes that all teeth receive replacement restorations prior to receiving root canal treatment or crowns, only after which they can be extracted [16, 19]. Whilst this may reflect the situation in Germany, this is not the situation in NHS primary care, where many restored teeth are extracted prior to receiving root canal treatment and many teeth with root canal treatment do not receive crowns, for example [20, 21]. Additionally, the patient fee and clinician remuneration do not vary based on the material choice in the English NHS system. Translating economic findings and lessons learnt from other countries which have phased out amalgam is therefore unwise.

The aim of this study was therefore to quantify the relative long‐term costs and consequences of amalgam versus composite direct posterior restorations in adult permanent teeth in the English NHS setting to help inform a potential amalgam phase‐out by 2030.

## 2 Methods

### 2.1 Perspective, Setting and Model

**FIGURE 1:**
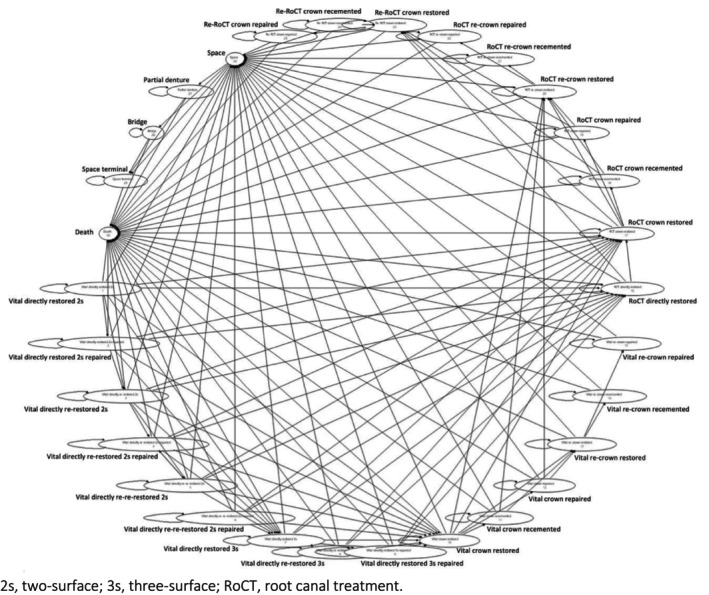
State transition diagram of reintervention following direct restoration placement. 2s, two‐surface; 3s, three‐surface; RoCT, root canal treatment.

An extended medical‐sector perspective, considering costs from patient and funder perspectives with extended societal considerations, in the English NHS primary care setting was taken. Class II amalgam restorations were compared with conventional, flowable bulk‐fill (f‐BF) and paste bulk‐fill (p‐BF) restorations. A microsimulation model of restoration failure and reintervention was iteratively constructed with input from clinical dental and economic experts to provide face validity and parameterised for amalgam in TreeAge Pro 2023–2024 (TreeAge Software). It was based on a review of the literature, primarily using a large, long‐term NHS claims dataset, which outlined and quantified the type of reintervention following failure of direct amalgam restorations, the proportion of root‐filled teeth which were restored with direct restorations or crowns, and the type of reinterventions following failure of crowns in the setting of interest [20, 21, 22, 23, 24]. The model states and possible state transitions (incorporating assumptions), including allocation probabilities (APs), are shown in Figure 1 and Table 1, alongside data used to inform these. Expert opinion was obtained from two clinical dental experts and two economic modelling experts.

**TABLE 1:** State‐to‐state transition probabilities.

State	Transition state(s)	Allocation probabilities	Data source
Vital unrestored carious lower left second premolar	2s	1	Expert opinion based on Bailey (2025) [25]
Vital directly restored 2 surface (2s)	re2s; 2sR; 3s; C; RoCTD; RoCTC; S	0.28; 0.11; 0.28; 0.08; 0.0888; 0.0312; 0.13	Expert opinion based on Lucarotti et al. (2014) [21]; Lucarotti et al. (2005) [20]
Vital directly re‐restored 2 surface (re2s)	re‐re2s; re2sR; 3 s; C; RoCTD; RoCTC; S	0.28; 0.11; 0.28; 0.08; 0.0888; 0.0312; 0.13	
Vital directly re‐re‐restored 2 surface (re‐re2s)	re‐re2sR; 3s; C; RoCTD; RoCTC; S	0.11; 0.56; 0.08; 0.0888; 0.0312; 0.13	
Vital directly restored 2 surface repaired (2sR)	re2s; 3s; C; RoCTD; RoCTC; S	0.28; 0.39; 0.08; 0.0888; 0.0312; 0.13	Expert opinion
Vital directly re‐restored 2 surface repaired (re2sR)	re‐re2s; 3s; C; RoCTD; RoCTC; S	0.28; 0.39; 0.08; 0.0888; 0.0312; 0.13	Expert opinion
Vital directly re‐re‐restored 2 surface repaired (re‐re2sR)	3s; C; RoCTD; RoCTC; S	0.67; 0.08; 0.0888; 0.0312; 0.13	Expert opinion
Vital directly restored 3 surface (3s)	3s; 3sR; C; RoCTD; RoCTC; S	0.56; 0.11; 0.08; 0.0888; 0.0312; 0.13	Expert opinion based on; Lucarotti et al. (2014) [21]; Lucarotti et al. (2005) [20]
Vital directly re‐restored 3 surface (re3s)	C; RoCTD; RoCTC; S	0.44; 0.0888; 0.0312; 0.44	
Vital directly restored 3 surface repaired (3sR)	C; RoCTD; RoCTC; S	0.44; 0.0888; 0.0312; 0.44	Expert opinion
Vital crown restored (C)	C; Crec; Crep; RoCTC; RoCTreC; S	0.15; 0.3; 0.15; 0.05; 0.05; 0.3	Expert opinion based on Burke and Lucarotti (2009) [22]
Vital crown recemented (Crec)	C; RoCTC; S	0.45; 0.1; 0.45	Expert opinion
Vital crown repaired (Crep)	C; RoCTC; S	0.45; 0.1; 0.45	
Vital re‐crown (reC)	reCrec; reCrep; S	0.3; 0.15; 0.55	
Vital re‐crown recemented (reCrec)	S	1	
Vital re‐crown repaired (reCrep)	S	1	
RoCT directly restored (RoCTD)	RoCTC; ReRoCTC; S	0.3; 0.18; 0.52	Expert opinion based on Lucarotti et al. (2014) [21]; Burke and Lucarotti (2009) [22]; Lumley et al. (2008) [23]
RoCT crown restored (RoCTC)	RoCTC; RoCTCrec; RoCTCrep; ReRoCTC; S	0.15; 0.3; 0.15; 0.3; 0.3	
RoCT crown recemented (RoCTCrec)	RoCTC; ReRoCTC; S	0.45; 0.1; 0.45	Expert opinion
RoCT crown repaired (RoCTCrep)	RoCTC; ReRoCTC; S	0.45; 0.1; 0.45	
RoCT re‐crown (RoCTreC)	RoCTreCrec; RoRCTreCrep; ReRoCTC; S	0.3; 0.15; 0.55	
RoCT re‐crown recemented (RoCTreCrec)	S	1	
RoCT re‐crown repaired (RoCTreCrep)	S	1	
Re‐RoCT crown restored (ReRoCTC)	ReRoCTCrec; ReRoCTCrec; S	0.3; 0.15; 0.55	
Re‐RoCT crown recemented (ReRoCTCrec)	S	1	
Re‐RoCT crown repaired (ReRoCTCrep)	S	1	
Space (S) (temporary, following extraction)	ST; B; PD	0.808; 0.176; 0.016	Expert opinion based on Adult Dental Health Survey 2009 (NHS Digital 2011) [24]
Space terminal (ST) (unrestored)	ST	1	
Bridge (B) (post‐extraction) (2 max)	B	1	
Partial denture (PD) (post‐extraction) (4 max)	PD	1	

Though amalgam and composites may fail in different ways and with differing incidences in patients with differing risk profiles, it is not currently clear from the limited evidence that there is a meaningful difference in re‐intervention mode following their failure [25]. It would therefore be inappropriate to complexify the model in terms of varying failure mode and reintervention for the different materials. The available reintervention data following amalgam restoration is therefore also used to structure the model and provide allocation probabilities for composite, assuming similar patterns of reintervention. An explanation of the model structure and terminology is provided elsewhere [25].

### 2.2 Model Outputs

The primary outcome measure obtained from the model was lifetime survival of the restored tooth. Secondary outcomes were: survival of the initial restoration; lifetime tooth survival limited to direct restorations; total treatment time; total number of treatment visits; and treatment time saved when using flowable bulk‐fill (f‐BF) and paste bulk‐fill (p‐BF) composites compared to conventional composite. Lifetime costs obtained from the model were: NHS funder's; NHS patient's; and clinician/practice laboratory costs.

### 2.3 Model Inputs

Because of the absence of NHS primary care data on the survival of class II composite restorations [16], the difference in survival of composite and amalgam restorations was calculated from randomised controlled trial data [26] as risk ratios (RRs), which were then applied to the baseline of reintervention following amalgam restoration in the NHS setting [27] (shown as transition probabilities (TPs) in Table 2). These sources were used as they reported survival for 2‐ and 3‐surface restorations separately, which other available data did not [13]. Assumptions of linear failure rates were made with a plan to validate outputs against NHS data to assess potential impacts. The RR approach [19, 32], assuming constant hazards, was also used to parameterise subsequent interventions as shown in Table 3 with data sources. There were no differences in survival between p‐BF, f‐BF, and conventional composites based on longer‐term (≥ 5 years) randomised clinical trials with *n* > 20 per group [33, 34, 35] therefore, survival outcomes did not change between composite materials.

**TABLE 2:** State transition probabilities.

State	Transition probability/6‐month cycle	Distribution	Data source
Vital unrestored carious lower left second premolar	1	—	Expert opinion based on Bailey (2025) [25]
Vital directly restored 2 surface^a^	0.028	Beta	Lucarotti and Burke (2018) [27]
Vital directly restored 2 surface repaired^a^	0.044	Beta	Lucarotti and Burke (2018) [27] and Gordan et al. (2015) [28]
Vital directly restored 3 surface^a^	0.034	Beta	Lucarotti and Burke (2018) [27]
Vital directly restored 3 surface repaired	0.054	Beta	Lucarotti and Burke (2018) [27] and Gordan et al. (2015) [28]
Vital crown restored^a^	0.020	Beta	Lucarotti and Burke (2018) [27]
Vital crown recemented^a^	0.020	Beta	Lucarotti and Burke (2018) [27]
Any vital crown repaired^a^	0.020	Beta	Lucarotti and Burke (2018) [27]
RoCT directly restored	0.044	Beta	Lucarotti and Burke (2018) [27] and Lucarotti et al. (2014) [21]
RoCT crown restored^a^	0.026	Beta	Lucarotti and Burke (2018) [27] and Lucarotti et al. (2014) [21]
RoCT crown recemented^a^	0.026	Beta	Lucarotti and Burke (2018) [27] and Lucarotti et al. (2014) [21]
RoCT crown repaired^a^	0.026	Beta	Lucarotti and Burke (2018) [27] and Lucarotti et al. (2014) [21]
Re‐RoCT crown restored	0.033	Beta	Lucarotti and Burke (2018) [27]; Lucarotti et al. (2014) [21] and Kwak et al. (2019) [29]
Re‐RoCT crown recemented	0.033	Beta	Lucarotti and Burke (2018) [27]; Lucarotti et al. (2014) [21] and Kwak et al. (2019) [29]
Re‐RoCT crown repaired	0.033	Beta	Lucarotti and Burke (2018) [27]; Lucarotti et al. (2014) [21] and Kwak et al. (2019) [29]
Bridge^a^	0.015	—	Burke and Lucarotti (2012) [30]
Partial denture^a^	0.032	Beta	Vermeulen et al. (1996) [31]

Abbreviation: RoCT, root canal treatment.

^a^ Includes replacement interventions of that restoration.

**TABLE 3:** Risk ratios to apply to baseline transition probabilities.

Relative intervention	Risk ratio	ln(RR) (se)	Distribution^a^	Data source
Composite vs. amalgam (2‐surface restoration)	2.05	0.72 (0.20)	Normal	Bernardo et al. (2007) [26]
Composite vs. amalgam (3‐surface restoration)	3.29	1.19 (0.35)	Normal	Bernardo et al. (2007) [26]
Composite vs. amalgam (combined restorations)	1.90	0.64 (0.11)	Normal	Worthington et al. (2021) [13]
Repair vs. replacement direct restoration	1.60	0.47 (0.14)	Normal	Gordan et al. (2015) [28]
Re‐RoCT vs. RoCT	1.30	0.26 (0.01)	Normal	Kwak et al. (2019) [29]
Direct restoration vital vs. RoCT	1.30	0.26 (0.01)	Normal	Lucarotti et al. (2014) [21]
Crown vital vs. RoCT	1.30	0.26 (0.03)	Normal	Lucarotti et al. (2014) [21]

Abbreviations: ln, natural logarithm; RoCT, root canal treatment; se, standard error.

^a^ Distribution sampled on log scale [32].

Patient and funder costs were based on NHS Unit of Dental Activity (UDA) values in 2023 English pounds and data on the proportion of paying/non‐paying patients accessing care [36, 37] as shown in Table 4.

**TABLE 4:** English NHS dental costs.

Treatment	NHS band	UDAs^d^	UDA fee (£)	Patient charge^e^ (payer) (£)	Patient charge (exempt) (£)	Average patient charge^b^, ^e^ (£)	Total NHS cost^c^ (payer) (£)	Total NHS cost^c^ (exempt) (£)	Average NHS cost^b^ (£)
Direct restoration (including repair crown) or extraction	2a	2	58.64	44.90	0	34.30	13.74	58.64	24.34
Root canal treatment ± direct restoration	2b	4	117.28	44.90	0	34.30	72.38	117.28	82.97
Any treatment involving an indirect restoration^a^	3	11	322.52	281.00	0	214.68	41.52	322.52	107.84
Recement crown (emergency)	4	1.2	35.18	25.80	0	19.71	9.38	35.18	15.47

Abbreviations: NHS, National Health Service; UDA, unit of dental activity 2023.

^a^ includes tooth‐borne fixed and removable prosthodontics (not implant‐borne).

^b^ Based on exempt 23.6%: payer 76.4% adult courses of treatment [36, 37] (example calculation for average NHS cost band 2a = [13.74 × 0.764] + [58.64 × 0.236] = £24.34).

^c^ Average UDA value £29.32 (BDA data, Diddee, personal communication, 2023).

^d^ With 1 UDA subtracted for non‐emergency treatments.

^e^ With band 1 charge subtracted for non‐emergency treatments.

Procedural time inputs and laboratory fees and visits associated with fixed and removable prosthodontic options used in the model are shown in Tables S1 and S2. Marginal time differences for p‐BF and f‐BF compared to conventional composite are shown in Table S3.

### 2.4 Analysis

The model extrapolated costs and outcomes of directly restoring a cariously‐cavitated lower left second premolar (to simplify results and avoid clustering effects) [19] in 10 000 18‐year‐old English NHS patients with initial mesio‐occlusal amalgam or composite restorations over a lifetime‐horizon with 6‐monthly cycles. Half‐cycle correction was applied alongside 3.5% discounting to costs and outcomes in the base‐case [38]. Individuals' probability of death changed with age at each subsequent cycle based on UK statistics of all‐cause mortality [39]. The base‐case scenario considered costs from an NHS funder's perspective. Deterministic and probabilistic sensitivity and scenario analyses were performed to characterise the uncertainty in the results and explore plausible parameter variations. This included using RRs and 95% confidence intervals for composite failure from meta‐analyses [13], using costs from alternative perspectives and non‐discounting for both costs and outcomes, which also provide internal validity checks by assessing their impact on the results. Further internal and external validity checks were carried out with the undiscounted model to compare against actual NHS restoration and tooth survival data [27]. Data distributions used with source references are shown in Tables 2 and 3, Tables S1 and S3, and deterministic analyses performed are shown in Table 4.

## 3 Results

### 3.1 Lifetime Costs and Outcomes

Over a lifetime, amalgam restored teeth dominated conventional composites, costing less to patients (£70) and funders (£34), surviving longer (4 years; 12 years non‐discounted), having increased survival until a direct restoration was no longer possible (6 years; 17 years non‐discounted), having reduced treatment time (43 min), having fewer treatment visits (1) and incurring lower laboratory costs to dentists or dental practices (£8) (Table 5, Table S4). These findings were robust with probabilistic and deterministic sensitivity analyses (Table 5). The main results of the probabilistic sensitivity analysis are shown in Figure S1, which shows the point estimates of each iteration of 1000 instances of following 10 000 patients through the model for base‐case NHS funder costs and tooth survival. The clear separation of the estimates for each intervention shows that amalgam was more effective and less costly than composite with negligible chance of uncertainty.

**TABLE 5:** Scenario and sensitivity analyses.

Scenario	Mean cost (£) (SD)		Mean tooth survival (years) (SD)		Mean treatment time (minutes) (SD)		Mean laboratory costs (£) (SD)	
	Amalgam	Composite	Amalgam	Composite	Amalgam	Composite	Amalgam	Composite
Base‐case (NHS costs)	81 (46)	115 (55)	21 (7)	17 (8)	64 (30)	107 (42)	11 (18)	18 (23)
Probabilistic base‐case	80 (0.5)	114 (0.6)	21 (0.1)	17 (0.1)	64 (0.3)	106 (0.4)	11 (0.2)	18 (0.2)
No discounting	147 (91)	181 (97)	44 (22)	32 (22)	110 (56)	156 (68)	25 (36)	35 (41)
NHS costs—all pay	43 (24)	60 (29)	21 (7)	17 (8)	64 (30)	107 (41)	11 (18)	19 (23)
NHS costs—none pay	201 (126)	290 (153)	21 (7)	17 (8)	64 (30)	106 (42)	11 (18)	18 (23)
Patient costs	120 (80)	175 (99)	21 (7)	17 (8)	64 (30)	107 (43)	11 (18)	18 (23)
Patient costs—all pay	158 (106)	228 (128)	21 (7)	17 (8)	64 (30)	106 (42)	11 (18)	18 (23)
Patient costs—none pay*	0 (0)	0 (0)	21 (7)	17 (8)	64 (30)	106 (41)	11 (18)	18 (23)
Composite RR upper bound unvarying (3.57)	80 (46)	130 (58)	21 (7)	15 (8)	64 (30)	119 (43)	11 (18)	22 (25)
Composite RR lower bound unvarying (0.9)	80 (46)	77 (45)	21 (7)	21 (7)	64 (30)	77 (34)	11 (18)	10 (17)
Composite RR unvaried by surfaces involved (1.89)	80 (46)	107 (53)	21 (7)	18 (8)	64 (30)	101 (39)	11 (18)	16 (22)
50% replace missing tooth	88 (50)	127 (58)	21 (7)	18 (8)	68 (32)	113 (42)	17 (24)	29 (30)
50% replace missing toothb^ND^	169 (101)	209 (103)	44 (22)	32 (22)	120 (61)	169 (71)	42 (50)	57 (51)
Extraction AP halved for vital direct restorations and equally applied to RoCT direct or RoCT crown restored	87 (49)	125 (55)	22 (6)	18 (7)	69 (32)	114 (42)	12 (17)	20 (22)
Extraction AP halved^ND^	164 (91)	201 (94)	47 (21)	35 (22)	120 (58)	170 (68)	28 (36)	38 (41)
Effectiveness outcome limited to direct restorations survival	80 (46)	114 (56)	18 (8)	12 (7)	64 (30)	107 (42)	11 (18)	18 (23)
Effectiveness outcome limited to direct restorations survival^ND^	147 (91)	180 (97)	35 (21)	18 (14)	109 (56)	156 (68)	25 (36)	34 (41)

*Note:* Amalgam—composite. Values rounded to nearest integer or one significant figure when < 1. All incremental cost effectiveness ratios (ICERs) negative other than *ICER = 0.

Abbreviations: AP, allocation probability; ND, no discounting; RoCT, root canal treatment; RR, risk ratio; SD, standard deviation.

Using data related to p‐BF and f‐BF rather than conventional composite resulted in modest changes to discounted time saving of 4 and 3 min respectively over a lifetime (Table S5).

### 3.2 Validation

Model data of restoration survival at 15‐years were the same as NHS data as shown in Figure S2 providing evidence for the internal validity of the model. The model slightly overestimates restoration survival initially, then potentially underestimates survival later. Tooth survival data from the model are very similar to NHS data over 15‐years [27] as shown in Figure S3, showing good external validity.

Scenario analyses resulted in expected cost and outcome changes in relation to the base‐case scenario providing further evidence of model internal validity (Table 5). For example, increasing the proportion of people receiving root canal treatment rather than extraction increased mean tooth survival and mean costs for interventions, and increasing the proportion of people replacing missing teeth had no effect on mean tooth survival but increased mean costs.

## 4 Discussion

When modelling costs and outcomes of different direct posterior restorations over a lifetime in English NHS primary care, amalgam was less costly to all stakeholders with better outcomes than conventional, f‐BF, and p‐BF composites for all outcomes modelled with very little uncertainty. There was a large increase in time until a direct restoration was no longer possible when amalgam was placed compared to composite, which is potentially important for tooth survival in those who cannot afford more expensive indirect restorations [40].

Previous economic evaluations of amalgam alternatives focus on restoration and or tooth longevity often measuring costs from a single perspective [18, 19]. This study expands the field by considering multiple perspectives and multiple relevant outcomes. Differences in treatment time and visits mean that patients incur higher indirect costs in terms of loss of productivity and these can be calculated as can differences in dental practice costs due to time. These may result in budget or access issues which are also important to funders [16]. When selecting a restoration, cost is by far the most important aspect of a direct restoration to patients [41]. They also value having reduced treatment time, waiting time, post‐operative complications, increased restoration longevity, and white over silvery‐grey restoration colour in terms of their willingness to pay. All of these except colour favour amalgam and these values also vary by income which additionally favour the use of amalgam in low‐income patients who commonly have increased treatment need [16, 41].

Comparing the model outputs with other recent models used to economically evaluate amalgam versus composite restorations shows broadly similar relative outcomes where comparable [18, 19], despite the differing settings, assumptions and limitations made with the previous and current models. This provides a level of external validity. One model did however report a difference in survival between conventional and bulk‐fill composites suggesting conventional composite was more cost‐effective [19]. Data with much shorter follow‐up and very few failures were used in that analysis however and this can be misleading [42]. The current analysis is based on longer‐term clinical data. Only restoration survival, tooth survival and limited costs have been modelled previously, so this analysis is broader in its scope. It also modelled a more realistic treatment pathway broadly based on data, which the previous models did not. The available NHS data was able to provide a level of external validity for the model, which has not been possible for other models.

Beyond the costs and outcomes measured in this study, composite and amalgam restorations also vary in many other ways which are important to patients, clinicians and funders [6, 16, 40, 41]. Failure to consider these when making implementation decisions can risk issues with patient uptake and access issues alongside unintended consequences [16]. Therefore the data presented here, alongside other relevant data, including indirect patient cost, dental practice cost, post‐operative complication, patient valuation, consumable cost and environmental data, for example, could be brought together as part of a more comprehensive economic evaluation in the form of a cost‐consequence analysis. This would allow the funder to consider all perspectives and weight them as it sees fit in coming to a decision on the feasibility of an amalgam phase‐out. Having said this, it is clear that an amalgam phase‐out will incur lifelong increased costs and treatment time to all parties with inferior outcomes and reduced access to care.

There are clear implications for policy makers. Remuneration needs to change as it is much lower than in the rest of Europe [16]. This has contributed to dentists leaving the NHS service and also the failure of NHS dentists to upskill to predictably place technically more demanding composite restorations [16]. Increasing the budget and/or limiting service provision must also be considered [16]. Education needs to improve, as though a large majority of UK clinicians have accessed postgraduate training on direct composites, this has not translated to them using recommended techniques (especially under NHS service) or a confident workforce when managing difficult situations with composite, especially when compared to amalgam [16, 40]. Enhanced training of NHS clinicians on direct posterior composites using documented techniques to deal with deep margins [43, 44] should be a priority in an attempt to reduce the differences in costs and outcomes as a phase‐out approaches. The need to reduce caries in the population by addressing modifiable risk factors is also critical to reduce the need for replacement and make publicly funded healthcare sustainable [16, 45].

### 4.1 Limitations

Whether the probabilistic sensitivity analysis represents the true uncertainty is questionable to a degree, as no representative data on composite survival in the English NHS setting exists. The dataset used to parameterise the base model of amalgam is very large, and therefore estimates are precise. The RRs for composite applied in relation to the base amalgam model have lower 95% CI bounds which are all much greater than 1 and therefore the separation seen in Figure S1 is perhaps unsurprising. It could be argued that the data are dated and comes from children who are often at higher risk of caries, questioning the validity of the results.

Despite these concerns, the Bernado study remains the most relevant to use because of the reasons already discussed, including the breakdown of survival data by surfaces restored, which is not included in other relevant studies [26]. It could also be argued that the Bernardo study is more representative of primary care than most randomised controlled clinical trials as higher risk individuals were not excluded (which they commonly are from many studies looking at restoration survival [42]). Equally, it could be suggested that this skews the RR in favour of amalgam survival relative to an adult population. Similarly studies comparing amalgam and composite tend to be a little older, as amalgam has barely been used in clinical trials in recent years, likely due to the planned phase‐out. The composite materials have likely improved since then, but the bonding agents have not. Fourth and fifth generation bonding agents pre‐date the trial and still have not been superseded by newer materials [46]. Having said that, newer materials are potentially less technique sensitive, which may make them more appropriate for use in primary care [47]. The Bernardo study stated that the same technical processes of restoring the cavities were used aside from the different materials, but the details were not provided, other than rubber dam was used ‘where possible’ [26]. It is very likely that circumferential matrix bands were used, rather than sectional bands which are now recommended for composite restorations [15, 48]. This is likely similar to the techniques used overwhelmingly in UK NHS primary care for placement of composite restorations, again favouring use of the Bernardo data [6, 16, 26]. Though composite materials have likely improved since the Bernardo trial, the materials themselves are generally of minor importance in restoration survival [45]. Supporting this, another study, originating from a similar time as the Bernardo study, shows excellent long‐term survival of large composite restorations, but critically when placed by a restorative expert often using multiple (including sectional) matrices per tooth, the results of which should therefore not be translated generally to primary care, as stated in the paper [14, 16].

Additionally, the RRs generated from meta‐analyses of randomised clinical trial data comparing all composite and amalgam restorations [13] and outcomes in the deterministic sensitivity analyses when using this data are not dissimilar to the base case.

It may be expected however, in a different setting where recommended techniques (e.g., sectional matrices and rubber dam) are commonly employed, composite is commonly used and remuneration is higher than the relatively low levels seen in the English NHS [16], that the uncertainty could increase.

A number of assumptions were made in constructing and parameterising the model, many of which have been stated. It was assumed that only one restoration was required whenever an intervention occurred, which might inflate actual costs. Old data were used from the setting of interest, alongside data from other settings with questionable relevance. There is a lack of NHS primary care reintervention data following composite or any restoration placement due to the absence of central data recording at the tooth level under the current UDA system. The model is based on restoration of a specific cavity in a specific tooth with questionable generalisability. Several parameters were estimated based on expert opinion, but these were not key parameters and sensitivity analysis suggested that these were of limited concern.

## 5 Conclusions

The model showed good internal and external validity, accurately predicting tooth survival following restoration in relation to long‐term NHS claims data. Amalgam was superior to conventional and bulk‐fill composites in terms of long‐term tooth survival, time until a direct restoration was required and costs for funders and patients. Treatment time, visits and laboratory fees to clinicians or dental practices for fixed and removable prosthodontics were also reduced over a lifetime. Without focussed educational change to upskill NHS clinicians and changes to remuneration and health service structure, an amalgam phase‐out in England will likely have significant lifelong impacts on restoration and tooth survival and costs for all stakeholders, whilst reducing societal productivity and exacerbating already existing issues of limited access to care and socio‐economic inequalities.

## Author Contributions

O. Bailey contributed to conception, design, data acquisition, analysis and interpretation, drafted and critically revised the manuscript. S. J. Stone contributed to conception, design, interpretation and critically revised the manuscript. G. Taylor contributed to design, data acquisition and critically revised the manuscript. L. Ternent contributed to conception, design, analysis and interpretation and critically revised the manuscript. C. R. Vernazza contributed to conception, design, data acquisition, analysis and interpretation and critically revised the manuscript. All authors gave their final approval and agree to be accountable for all aspects of the work.

## Ethics Statement

A favourable ethical opinion was obtained from Newcastle University Ethics Committee reference 7262/2018.

## Conflicts of Interest

The authors declare no conflicts of interest.

## Supporting information


**Data S1:** cdoe70050‐sup‐0001‐Supinfo.docx.

## Data Availability

The data that support the findings of this study are available from the corresponding author upon reasonable request.
