# Supplementary material for: Model‐Based Cost‐Effectiveness of Direct Restorations: Amalgam Dominates
Source: Community Dent Oral Epidemiol. 2025 Dec 21;54(4):407–17. doi: 10.1111/cdoe.70050 (PMC13428050; doi:10.1111/cdoe.70050)
Supplement: Supplementary file 1 — Data S1: cdoe70050‐sup‐0001‐Supinfo.docx. [file CDOE-54-407-s001.docx]

**Model-Based Cost-Effectiveness of Direct Restorations: Amalgam Dominates**

Bailey O, Stone S, Taylor G, Ternent L, Vernazza C.

**Supporting Information**

**Supporting Information Figures**

Figure S1. Probabilistic sensitivity analysis cost-effectiveness scatterplot


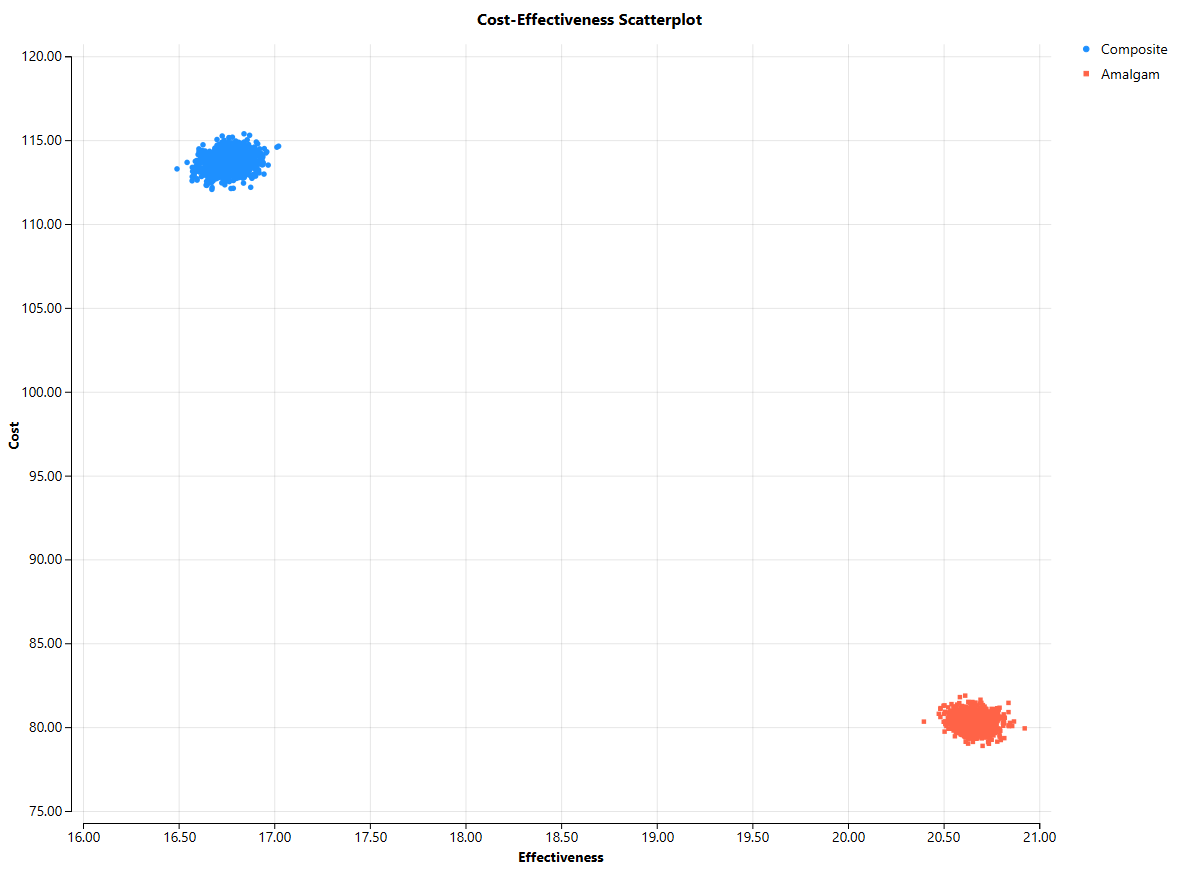


Effectiveness in years; cost in English pounds.

Figure S2. Premolar 2-surface amalgam restoration survival

NHS data from Lucarotti and Burke 2018.

Figure S3. Premolar tooth survival following a 2-surface amalgam restoration

NHS data from Lucarotti and Burke 2018.

**Supporting Information Tables**

Table S1. Appointment time booked, and private fee charged for mesio-occlusal (MO) premolar and mesio-occluso-distal (MOD) molar restorations

| **Restoration** | **Material** | **Appointment time booked (minutes)** | | | | |
| --- | --- | --- | --- | --- | --- | --- |
|  |  | Mean | SD | Range | Distribution | 95% CI |
| 2-surface MO premolar | Composite | 34 | 9 | 15 – 90 | Gamma | 34 – 34 |
|  | Amalgam | 24 | 7 | 10 – 60 | Gamma | 24 – 24 |
| 3-surface MOD molar | Composite | 42 | 11 | 15 – 120 | Gamma | 42 – 43 |
|  | Amalgam | 29 | 8 | 5 – 60 | Gamma | 29 – 30 |

Data from Bailey et al. 2022 and Bailey 2025. SD, standard deviation; CI, confidence interval.

Table S2. Intervention events (other than 2 and 3 surface restorations) average number of visits, treatment time and lab bill for English NHS provision

| **Procedure (lower second premolar)** | **Average number of visits (range)** | **Average treatment time (minutes) (range)** | **Average lab bill (£) (range)** |
| --- | --- | --- | --- |
| Crown | 2 | 50 (45-60) | 32.33 (30-35) |
| RoCT | 1 | 38.33 (30-45) | N/A |
| RoCT + direct restoration | 1 | 46.67 (40-60) | N/A |
| RoCT + crown | 2 (2-3) | 83.33 (75-100) | 32.33 (30-35) |
| Re-RoCT | 1 (1-2) | 51.57 (45-60) | N/A |
| Re-RoCT + crown | 3 (2-4) | 96.67 (90-110) | 32.33 (30-35) |
| Extraction | 1 | 23.33 (20-30) | N/A |
| Partial denture | 4 (3-4) | 50 (30-60) | 90 (70-120) |
| Resin bonded bridge | 2 | 50 (45-60) | 53.33 (45-65) |
| Conventional bridge | 2 | 50 (45-60) | 68.33 (65-70) |
| Bridge: average* | 2 | 50 (45-60) | 66.23 (45-70) |
| Recement crown | 1 | 16.67 (15-20) | N/A |
| Repair crown | 1 | 16.67 (15-20) | N/A |
| Direct restoration repair | 1 | 16.67 (15-20) | N/A |

Based on expert opinion (n=3). RoCT, root canal treatment; N/A, not applicable; *Based on 86% conventional bridge and 14% resin bonded bridge provision under NHS regulations (Burke and Lucarotti 2012).

Table S3. Marginal time differences for varying composite restorations

| **Composite material** | **Restoration surfaces involved** | **Extra time (minutes)** | **Distribution** | **Data source** |
| --- | --- | --- | --- | --- |
| Conventional vs bulk-fill flowable | 2 | 1.74 | None* | Extrapolation Bailey et al. 2022 and Güler and Karaman 2014 |
|  | 3 | 2.17 | Gamma | Güler and Karaman 2014 |
| Conventional vs bulk-fill paste | 2 | 2.33 | None* | Extrapolation Bailey et al. 2022 and Güler and Karaman 2014 |
|  | 3 | 2.91 | Gamma | Güler and Karaman 2014 |

*No genuine distribution exists due to the estimate derivation from data extrapolation.

Table S4. Base-case scenario 3.5% discounting

| **Statistic** | | **Cost (£)** | | **Tooth survival (years)** | | **Treatment time (minutes)** | | **Treatment visits** | | **Laboratory costs (£)** | |
| --- | --- | --- | --- | --- | --- | --- | --- | --- | --- | --- | --- |
|  |  | **Amalgam** | **Composite** | **Amalgam** | **Composite** | **Amalgam** | **Composite** | **Amalgam** | **Composite** | **Amalgam** | **Composite** |
| Mean | | 81 | 115 | 21 | 17 | 64 | 107 | 3 | 4 | 11 | 18 |
| Standard deviation | | 46 | 55 | 7 | 8 | 30 | 42 | 1 | 1 | 18 | 23 |
| Quantiles | Minimum | 24 | 24 | 0 | 0 | 8 | 18 | 1 | 1 | 0 | 0 |
|  | Median | 68 | 110 | 23 | 18 | 59 | 103 | 2 | 3 | 5 | 14 |
|  | Maximum | 402 | 413 | 28 | 28 | 252 | 351 | 13 | 13 | 227 | 227 |
| Prediction interval | 95% lower bound | 80 | 114 | 21 | 17 | 64 | 106 | 3 | 3 | 10 | 18 |
|  | 95% upper bound | 81 | 116 | 21 | 17 | 65 | 108 | 3 | 4 | 11 | 19 |

Values rounded to nearest integer or one significant figure when <1.

Table S5. Marginal time differences for composite material variations. Base-case with and without 3.5% discounting and probabilistic sensitivity analysis

| **Statistic** | | **Time saving for bulk-fill compared to conventional layered composite (minutes)** | | | | | |
| --- | --- | --- | --- | --- | --- | --- | --- |
|  |  | **Bulk-fill flowable** | | | **Bulk-fill paste** | | |
|  |  | 3.5% discounted | No discounting | Probabilistic sensitivity analysis | 3.5% discounted | No discounting | Probabilistic sensitivity analysis |
| Mean | | 3 | 4 | 3 | 4 | 5 | 4 |
| Standard deviation | | 2 | 2 | 0.02 | 2 | 3 | 0.02 |
| Quantiles | Minimum | 2 | 2 | 3 | 2 | 2 | 4 |
|  | Median | 3 | 4 | 3 | 4 | 5 | 4 |
|  | Maximum | 9 | 12 | 3 | 12 | 15 | 4 |
| Prediction interval | 95% lower bound | 3 | 4 | 3 | 4 | 5 | 4 |
|  | 95% upper bound | 3 | 4 | 3 | 4 | 5 | 4 |

Values rounded to nearest integer or one significant figure when <1

**References**

Bailey O. 2025. Amalgam phase-out: what next for dentistry? The costs and benefits of the alternative direct restorations [dissertation]. [Newcastle upon Tyne (UK)]: Newcastle University. [accessed June 5, 2025]. <https://theses.ncl.ac.uk/jspui/handle/10443/6481>

Bailey O, Vernazza CR, Stone S, Ternent L, Roche AG, Lynch C. 2022. Amalgam phase-down part 1: UK-based posterior restorative material and technique use. JDR Clin Trans Res. 7(1):41-49.

Burke FJT, Lucarotti PSK. 2012. Ten year survival of bridges placed in the General Dental Services in England and Wales. J Dent. 40(11):886-95.

Güler E, Karaman E. 2014. Cuspal deflection and microleakage in premolar teeth restored with bulk-fill resin-based composites. Journal of Adhesion Science and Technology. 28(20):2089-2099.

Lucarotti PSK, Burke FJT. 2018. The ultimate guide to restoration longevity in England and Wales. Part 7: Premolar teeth: Time to next intervention and to extraction of the restored tooth. Brit Dent J. 225(7):633-644.
